# Hybrid flexible ureteroscopy strategy in the management of renal stones – a narrative review

**DOI:** 10.25122/jml-2022-0110

**Published:** 2022-08

**Authors:** Bogdan Geavlete, Cristian Mareș, Răzvan Mulțescu, Dragoș Georgescu, Petrișor Geavlete

**Affiliations:** 1 Department of Urology, Sanador Hospital, Bucharest, Romania; 2 Department of Urology, Emergency Clinical Hospital Sfântul Ioan, Bucharest, Romania

**Keywords:** flexible ureteroscopy, single-use, reusable, hybrid technique, renal stones

## Abstract

The introduction of single-use flexible ureteroscopes (suFURSs) in daily practice tends to overcome the main limitations of reusable ureteroscopes (reFURSs), in terms of high acquisition costs, maintenance, breakages and repairing costs, reprocessing and sterilization, as retrograde intrarenal surgery (RIRS) is promoted as first-line treatment of renal stones in most cases. A hybrid strategy implies having both instruments in the armamentarium of endourology and choosing the best strategy for cost-efficiency and protecting expensive reusable instruments in selected high-risk for breakage cases such as large stones of the inferior calyx, a steep infundibulopelvic angle or narrow infundibulum, or abnormal anatomy as in horseshoe and ectopic kidney. In terms of safety and efficiency, data present suFURSs as a safe alternative considering operating time, stone-free, and complication rates. An important aspect is highlighted by several authors about reusable instrument disinfection as various pathogens are still detected after proper sterilization. This comprehensive narrative review aims to analyze available data comparing suFURSs and reFURSs, considering economic, technical, and operative aspects of the two types of instruments, as well as the strategy of adopting a hybrid approach to selecting the most appropriate flexible ureteroscope in each case.

## INTRODUCTION

Nephrolithiasis is one of the most frequent pathologies affecting the urinary tract, with a worldwide incidence of 12%, affecting more than half-million patients only in the United States yearly. The incidence of urinary stones has doubled in the last decades, associated with an increased risk of recurrence that rises to 50% ten years from the first episode [1, 2]. It affects both sexes, with a higher incidence in male patients, especially between the 2^nd^ and 4^th^ decade of life [3, 4]. Studies suggest that the constantly increasing incidence is mainly related to lifestyle modifications such as dietary habits and physical activity [5, 6]. Other risk factors involved in the etiology of urinary lithiasis are related to genetics, geographical localization due to warmer temperatures leading to dehydration, water intake volume, body weight, various comorbidities such as diabetes mellitus or iatrogenic factors [7].

The heterogeneity of urinary lithiasis is diverse, implying various stone compositions, dimensions, or localization along the urinary tract. When selecting the strategy for treatment, multiple variables are considered, such as stone characteristics (location, size, number, and composition), clinical factors, and renal anatomy. The European Association of Urology clearly defines guideline recommendations for interventional treatment of renal stones as follows: for stones larger than 20 mm, percutaneous nephrolithotomy (PCNL) is the first treatment of choice, whereas, for stones smaller than 10 mm, extracorporeal shock-wave lithotripsy (ESWL) is the preferred option. In all other cases or when the failure of the other treatment modalities was encountered, retrograde intrarenal surgery (RIRS) is the new “gold standard” of endourology in stone management [8]. A particular case is represented by the lower pole of the kidney, where variable negative factors, such as closed infundibulopelvic angle, narrow infundibulum (<5 mm), or long infundibular length, could represent first-line treatment in performing RIRS [9].

Flexible ureteroscopy (FURS) was first pioneered by Marshal in 1964 [10] and has been used successfully ever since for upper tract urinary lithiasis with stone-free rates (SFRs) up to 90% [11]. Bagley introduced disposable ureteroscopes in 1987 [12], and their popularity has constantly grown [13, 14], imposing comparable results to those of reusable ureteroscopes in technical specificities such as deflection, optics or irrigation flow [15, 16]. Many studies have been conducted recently, comparing the two instruments, highlighting the better operative time, good SFR, or complications rate for single-use FURS (suFURS) [17], while the reusable FURS (reFURS) performed better in terms of image quality and maneuverability [18, 19].

In order to limit the main inconvenience of reFURS such as high acquisition cost – up to $25,000 with an additional cost of viewing monitor and video processor [20], maintenance costs up to $100,000, yearly [21], breakages and reprocessing between procedures, the introduction of the new suFURSs tends to overcome all these hidden costs while preserving efficacy and technical advantages of the reusable scopes. Regardless of the general effectiveness of FURS, the relatively high cost of reusable ureteroscopes and their issues of durability still represent the burden of its acquisition, especially in developing countries [22]. Many recent studies overviewed a comparison of cost-efficiency between reusable and single-use FURS [23–26], concluding that only high-volume centers could benefit from the use of reFURS on a daily basis, a statement that undoubtedly depends on the acquisition cost of suFURS [27].

Considering these aspects, the hypothesis of utilizing suFURS in high risk for breakage (HRFB) cases where the risk of damaging the expensive reFURS is increased, recent studies analyzed the feasibility of alternating between the two technical choices of endourological treatment [28–30]. This hybrid approach aims to create a strategy for choosing between reusable and single-use instruments for a better cost-efficiency ratio to select specific cases that are advantageous in utilization for each type of FURSs mentioned above. Therefore, the need for this review is imperative in comparing data on cost-efficiency for both instruments and highlighting cases that increase the risk of damaging reFURSs. The latest studies in this field were overviewed to analyze a potential hybrid strategy approach in selected cases of renal stones.

### Cost-efficiency comparison – suFURS *vs.* reFURS

**Figure 1:**
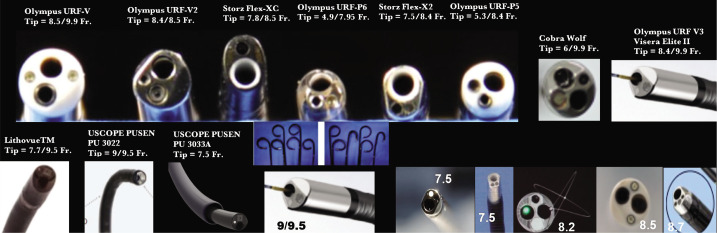
Common types of reFURS and suFURS used in daily practice.

**Figure 2:**
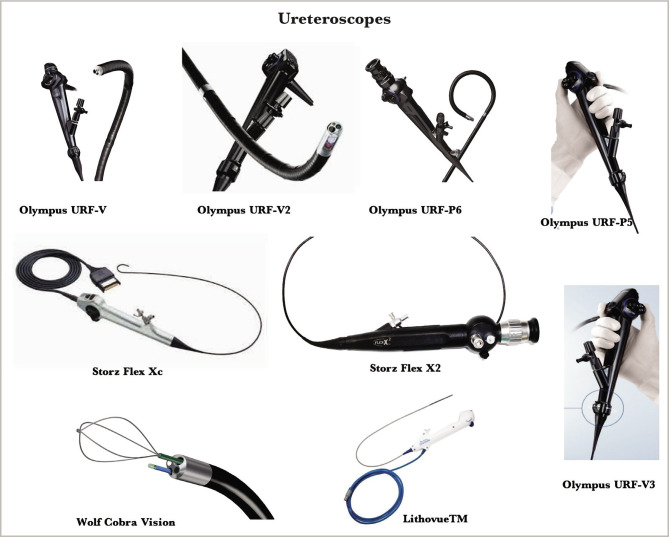
Some of the main types of reFURS and suFURS used in our experience.

This review is also based on our experience (over 35 years, more than 7.000 of such procedures) in which we used the majority of known reFURS and suFURS (Figures 1 and 2).

The introduction of suFURS in daily practice aimed to overcome the disadvantages of reFURS in various aspects of expenditure, damages of the instrument, and reprocessing while maintaining the quality and accessibility for the clinician. The overall cost of using reFURSs is highly dependent on the initial price of the instrument, as well as maintenance, sterilization, and costs of repairing. Many studies were conducted comparing the two types of instruments in terms of cost-efficiency, considering multiple variables such as cost of initial purchase, number of procedures for reFURSs until damages occur and repairs are required, as well as the cost of repair and sterilization.

The results of a recent systematic review from Michele Talso [31], including 19 studies of cost-efficiency between suFURS and reFURS, highlighted that despite the decreasing price of suFURS, the reusable ones are still more cost-effective when a high volume of procedures are conducted, even when considering all additional costs. It shows that the number of uses before the need for repair varies widely between 8 to 29 procedures, with an average cost per procedure between $120 and $1,212. Also, a significant variation in the number of total procedures was encountered between 14 and 643 operations. The study concluded that the cost of reFURS is indirectly proportional to the procedures/year in each center, underlining that high-volume centers benefit more from reusable endoscopes.

In recent years, many studies followed to determine the efficiency of the reFURS compared to the single-use scopes, showing that multiple factors are encountered in terms of longevity of ureteroscopes, such as the experience of the surgeon maneuvering the instrument, the number of surgeons having access to the same scope, type of the hospital – private *vs*. university and the experience of the personnel involved in maintenance and repair [32]. Alfane JS *et al*. suggest that the median period of a ureteroscope before requiring repair is 21 procedures [33], and performing surgeries by the same experienced surgeon, the longevity of the ureteroscope can reach up to 159 cases [34]. In 2014, a prospective study from Carey RI *et al*. [35] highlighted the importance of repairing instruments by the original company, rather than by a third-party source, with a mean value of 11, compared to 7, respectively. A cost analysis evaluating 655 procedures from 2016 shows that the cost of repairing varies widely from $233 to $7,521, with a mean value of $355 per repairing procedure [36]. A recent prospective study considering both repairing and acquisition costs of ureteroscopes shows that the price per procedure varies from $1,212 and $1,743 for the reFURS and $1,300 and $3,180 for suFURS [11]. An analysis of micro-costing by Kazumi-Taguchi [37], following all costs of acquisition, scope repair cost, recycling and reprocessing cost of labor and consumables, shows that the total expenditure for suFURS and reFURS were $2,852 and $2,799, underlying that despite the higher cost of acquisition of suFURS, the overall price when considering consumables, labor and repair costs of the two ureteroscopes was comparable.

The acquisition price of a new reFURS varies widely, depending on several factors, such as type of instrument, generation, or manufacturer brand. A conventional instrument from the German brand Karl Storz varies around $15,000 (Flex X ureteroscope) [38], while a digital scope from Olympus (URF-V) has a purchasing price of over $20,000 [39]. Studies suggest that reprocessing costs, including sterilization, consumables for packaging, and auxiliary personnel labor, are difficult to evaluate critically but would range between $20 to over $100 per procedure [11, 40], while costs of repair and the variability of procedures until a repair is required have been previously discussed. In terms of suFURS purchasing prices, the acquisition market is highly dependable on the instrument brand; prices vary from $700 for Polyscope [41] to, $800 for SemiFlex [42] and up to $1,300–$3,000 for LithoVue from Boston Scientific [43]. It is expected that the more brands enter the market with a new product, the prices are expected to decrease.

A study from Christopher J Martin [44] prospectively following a cost-benefit analysis on reFURS highlighted that after excluding the initial acquisition cost of the instrument, the amortized cost per procedure was around $850, calculating an overall financial benefit of using the reFURS in the detriment of suFURS after 99 procedures. He also concluded, as the authors above, that high volume centers are the only beneficiaries of using a reusable instrument, while hospitals with fewer procedures/year would benefit more from using suFURS.

### High risk for breakage cases and alternative treatments in abnormal kidneys

Flexible ureteroscopes have suffered important engineering processes in the last three decades in terms of up-grading and miniaturization. These technological advancements significantly improved instruments by enhancing maneuverability, visibility, and ergonomics, but they come with the expense of ureteroscope fragility, leading to significant expenses in high-volume centers. Damages of ureteroscopes can occur in non-operative conditions, such as sterilization, cleaning, or reprocessing between procedures and intra-operative events that occur by handling the FURS or using various accessory equipment such as laser fibers or baskets [45]. Sung JC *et al*. highlighted the most frequent locations of damaging events in handling a reFURS requiring repairs, as follows: impaired deflection components, working channel damage, eyepiece components damage, and scope shaft malfunction [46].

The most fragile part of the FURS is the active deflection in front of the ureteroscope, as deflection mechanisms, angulation cables, or bending sheath are fragile components and can be damaged in improper handling [47] especially in difficult conditions of accessing renal stones. Unlike the other mechanisms of breakage, predicting risks of over-deflecting FURSs during surgeries and using a suFURS instead of reFURS can lead to fewer incidences and the necessity of repair. A recent paper aiming to detect risk factors for damaging a FURS [48] suggests that a steep infundibulopelvic angle of less than 60° can lead to ureteroscope's breakage, considering the need for excessive deflection in managing a stone in that position for an extended period of time. It was demonstrated that relocating the stone in a more linear position, such as the upper pole or renal pelvis, and continuing fragmentation at that level can significantly reduce the risk of ureteroscope breakage [49]. However, relocating a stone is not always possible; if the stone is too large, generally over 10 mm, or calyceal stenosis is present, the surgeon is constrained to work in an over-deflected position, altering the ureteroscope [50]. A recent paper by Wilson Molina *et al*. [51] aimed to compare the use of suFURS instead of reFURS, especially in these high risk-for-breakage cases, to protect the expensive reFURS and to limit the number of necessary repairs in a cost-efficient model. The study resulted in 17 cases considered high risk-for-breakage, and suFURS were used from a total of 228 cases over 15 months, resulting in cost savings of approximately 5% of the total costs of ureteroscopy in this particular center, leading to total savings of approximately $9,500. It concluded that suFURS helps reduce the overall cost of ureteroscopy when used in cases of great difficulty that burden the reFURS, reducing frequent damages.

Renal calculi in kidney malformation represent a true challenge for every urologist; the most common renal fusion anomaly is horseshoe kidney (HSK), with an overall prevalence of 0.25% in the general population [52]. If open surgery was the elected treatment of such anatomical malformations in the past, with the advancement of technology and miniaturization of ureteroscopes, FURSs represent a valid alternative in the armamentarium of treating this type of lithiasis [53]. This type of minimally invasive surgery is associated with a reduced risk of perioperative complications while preserving high stone-free rates. A recent paper [54] compared the efficacy of suFURS and reFURS in treating HSK stones, following two similar groups of patients using a suFURS from Pusen Medical Technology and a reFURS URF-V2 from Olympus. After analyzing the operation time, mean stone burden, stone-free rate, and complications that followed the two treatment modalities, it concluded that flexible ureteroscopy is a safe and effective alternative treatment, with better results when using suFURS. Moreover, accessing the collecting system in a retrograde manner is not easy; it is associated with a ureteral insertion into the renal pelvis in a more superior and lateral position than a normal kidney; thus, more deflection is needed consecutively altering the ureteroscope [55].

Ectopic kidneys also represent a common abnormality of the reno-urinary system, and various conditions such as nephrolithiasis or hydronephrosis are frequent findings in patients presenting this type of malformation. Because of the abnormal position of the kidney, managing renal stones in these patients may be challenging. Thus, flexible ureteroscopy can overcome these limitations. Considering the advantages of active deflection, it can be an alternative in this particular case, with studies suggesting that stone-free rates can be achieved in up to 85% [56–58]. Nonetheless, the specific anatomy in ectopic kidneys implies malrotated kidneys and tortuous ureters that lead to increased difficulty in the surgical procedure, and over-deflection can occur, damaging the ureteroscope. A recent retrospective analysis [59] that followed 11 patients with ectopic pelvic kidneys treated with suFURS for renal stones underlined that disposable ureteroscopes represent a good alternative to reusable instruments in all parameters that were considered – stone-free and complication rates, as well as mean hospitalization period while decreasing the risk of damaging the reFURS in these challenging cases.

### Safety and efficacy of suFURS vs. reFURS

Continuous endourology and laser technology improvements implemented flexible ureteroscopy as a safe and effective method of minimally-invasive treatment for renal stones. The introduction of disposable flexible ureteroscopes promises comparable results with the reusable instruments in terms of operative time, stone-free, and complication rates [17]. Considering the image quality and the deflective mechanism, studies suggest that no significant differences were observed between reFURS and suFURS [16, 60].

A meta-analysis was performed last year by Yongchao Li *et al*. [61] in order to assess the differences between suFURS and reFURS. In terms of efficacy, suFURS was significantly associated with an increased stone-free rate compared to the reusable ureteroscope, while for perioperative complications, the two types of ureteroscopes performed similarly. The only aspect where suFURS had lower performance was total operative time, where reFURS was associated with a decreased duration of surgery. In a recent prospective multi-center study [62] following 180 patients treated with either suFURS or reFURS, no significant differences were detected for stone-free rates in both groups. However, perioperative complications in reFURS were 8.8%, compared with 3.3% in the suFURS group, and the first group also presented more major complications Clavien score III-V. In terms of postoperative infection rates, the reFURS presented a 16.6% rate of infectious events, compared to 3.3% in the disposable ureteroscope group. It concluded that suFURS has a comparable cost and stone-free rates while performing better in perioperative complications and infection rates. A systematic review and meta-analysis of the literature were performed last year by Chunyang Meng *et al*. [63], evaluating the stone-free rate, mean operative time, blood loss, and complication rates between the two endoscopic approaches with no significant differences observed between the two groups. Nonetheless, Manint Usawachintachit *et al*. [64] published a prospective study comparing 115 cases of suFURS and 65 cases of reFURS, underlining a significant decrease in overall operating time in suFURS *vs*. reFURS, including stone extraction. It concluded that suFURS is a feasible alternative to reFURS.

A comparative *in-vitro* analysis that aimed to determine differences in terms of deflection, visual capabilities, and irrigation flow of the current reFURS and suFURS highlighted several differences between instruments, such as better deflection and irrigation flow for suFURS, while reFURS presented better image quality compared to the disposable counterparts. At the end of the tests, several defects were observed for the disposable FURS, while reFURS presented no damages [65]. In a prospective study by Jonathan Kam *et al*. [19], 141 patients were followed for flexible ureteroscopy to assess renal stones, demonstrating that suFURSs perform similar to reFURSs and clinical outcomes were comparable to their more expensive versions. In a prospective multicenter study, Shiyoung Qi *et al*. [66] compared the efficacy and safety of using suFURS and reFURS in treating 126 patients for renal stones. A high-quality image was noted for both instruments, combined with similar operating time, length of hospital stay, and postoperative complications, concluding that suFURS were a safe and effective alternative to the reusable ureteroscopes.

An important issue of reFURS in terms of safety is suggested by some authors regarding the lack of proper decontamination of the instrument between procedures; data underlines that these events occurred even when high-level disinfection was performed. A recent study by Legemate *et al*. [67] showed that after collecting microbiological probes post-disinfection of reFURSs, 12% of them were positive, concluding that the incidence of urinary tract infections could be higher with the use of reusable instruments. Even when manual decontamination combined with hydrogen peroxide gas was performed, contamination of reFURS was still present, leading to cross-contamination between patients [68]. In terms of the intrinsic safety of the instrument, a prolonged period of cleaning and disinfection could lead to damage to the reFURS, considering the extremely fragile equipment. At the same time, suFURS eliminates all these risks of reprocessing and contamination between procedures [13].

A literature review that aimed to determine cross-contamination between procedure and sterilization process emphasized a general issue for flexible endoscopes such as cystoscopes, ureteroscopes, bronchoscopes, and duodenoscopes, which is that improper cleaning and decontamination leads to persistent bacteria on different parts of instruments. Pseudomonas aeruginosa was the most frequent pathogen, followed by *Klebsiella spp*., *Mycobacterium*, and *Escherichia coli*. It recommends competency training for personnel in charge of this responsibility so that further events of incomplete decontamination of reusable endoscopes would not occur [69]. These pathogens are a common finding in urinary tract infections, with an increased incidence in both male and female patients presenting a high variability in terms of overall bacterial resistance and multidrug resistance to common antibiotic treatment [70–72].

## Discussion

Considering the increasing incidence of urolithiasis, flexible ureteroscopy represents a key procedure in the treatment of renal stones. reFURSs have a high acquisition price and, combined with maintenance, sterilization, reprocessing, and repair costs may burden the economic impact of modern surgery in the endourological field. Different aspects have been discussed considering the advantages and disadvantages of using either a reusable ureteroscope or a disposable one, considering that a suFURS has no other hidden cost except the purchasing price. In addition, no labor in sterilization or reprocessing is further needed, no overloading for the auxiliary personnel, and no repairs are required when damages to the scope are encountered. The literature provided comparable results regarding the ureteroscope's function when maneuverability, visualization, operating time, stone-free rate, and postoperative complications were compared between the two types of instruments [17, 18, 65, 66].

The idea of promoting a hybrid approach of flexible ureteroscopy in the management of renal stones is not to determine which kind of instrument generally has better performances and a ubiquitous choice to be taken but to determine rationality in selecting cases in which suFURS can be performed, protecting the expensive reFURS form damages and further repairs while maintaining a favorable ratio in the economic pathway. This approach is new, and few data are reported to date. In the mid-summer of last year, Eugenio Ventimiglia *et al*. conducted a retrospective study [73] at Tenon Hospital in Paris, France, analyzing the benefit of introducing suFURS in managing difficult cases (both stone disease and malignant pathology of the upper urinary tract) to improve the damaging rates of reFURS and prolonging its life, comparing the two types of instruments (Table 1). They observed that by introducing suFURSs in high-risk cases, the longevity of reFURS was prolonged by 40%. The team considered high-risk for breakage situations such as stones of the lower pole, a steep angle between lower calyx and renal infundibulum, and complex anatomy of the reno-urinary system.

**Table 1:** Technical characteristics and acquisition prices of reFURSs and suFURSs [73].

Name	Brand	Width	Working channel	Imaging	Purchase cost
Technical characteristics and acquisition prices of reFURSs					
**URF-V3**	Olympus	8.4 Fr	3.6 Fr	Digital	$20,200–80,000
**Flex-X2**	Storz	7.5 Fr	3.6 Fr	Fiberoptic	$13,611–14,300
**Flex-Xc**	Storz	8.5 Fr	3.6 Fr	Digital	$17,425–70,390
**Cobra**	Wolf	9.9 Fr	3.6 Fr/2.4 Fr	Digital	$58,000
Technical characteristics and acquisition prices of suFURSs					
**Lithovue**	Boston Scientific	7.7 Fr	3.6 Fr	Digital	$1,300–3,180
**Uscope PU3022**	Pusen	9 Fr	3.6 Fr	Digital	$800

Data is consistent with the studies mentioned above that one of the most important factors in breaking the ureteroscope is a steep infundibulopelvic angle of less than 60° [48], and the attempt to relocate the stone from the lower pole to a more affordable position could reduce this risk [49]. In a recent systematic review, Sulaiman Sadaf Karim *et al*. [74] also suggest that managing a stone located in a steep infundibulopelvic angle that results in over-deflection could damage the ureteroscope, and other modalities of treatment should be considered, including a disposable ureteroscope. suFURS is also recommended by Bhaskar K. Somani *et al*. [75] in large renal stones or lower pole renal stones greater than 1 cm and when abnormal anatomy of the renourinary system is encountered. Similar data on using suFURS when managing stones located in anatomical variants of the kidney were previously presented by Geavlete B. *et al*. [54, 59] in horseshoe kidney and ectopic pelvic kidney, concluding that using a disposable ureteroscope in such cases may improve general outcomes.

Disposable ureteroscopes have been generally accepted in the armamentarium of most endourologists. Their qualities, similar to that of the reusable instruments, have been implemented in routine practice. A worldwide survey on the use of suFURS has highlighted that almost half of the interviewed urologists use a disposable ureteroscope regularly, and almost three-quarters of them specifically admitted they manage difficult cases in such manner [76]. A cost-efficiency problem was raised, questioning the profitability of disposable ureteroscopes and whether it was better to adopt a hybrid strategy using both types of instruments. Last year, Fanny Monmousseau *et al*. [77] conducted a study to compare the economic burdens of implementing a hybrid strategy mixing the usage of both suFURS and reFURS. The study model included hospital volume, costs of sterilization, and reprocessing. It concluded that adopting a hybrid strategy was cost-efficient, directly dependent on the number of surgeries, and using a predicting model in advance is beneficial in adopting this strategy. This data confirms the previously presented comparison between the cost-efficient strategy of using suFURS [31, 44] that suggested that only high-volume centers would benefit from using reFURS exclusively. The same results were obtained by Dries Van Compernolle *et al*. [78] in a retrospective cost-efficiency analysis after 983 cases of both single and reusable flexible ureteroscopes. He concluded that a hybrid strategy of using both instruments is more efficient, especially when using suFURS in high-risk for breakage cases, and the economic impact of using exclusively reFURS is diminished after more than 155 cases. It is generally accepted that further studies are needed to sustain the idea of adopting a hybrid flexible ureteroscopy strategy in managing renal stones, but preliminary data are consistent with these findings. The main limitation of this review is represented by the lack of a systematic review. However, little data is available on this particular topic, considering the hybrid strategy of using both suFURS and reFURS in treating renal stones as a novel concept of endourology.

## Conclusions

Flexible ureteroscopy represents an essential tool for every urologist managing renal stones. suFURS has become widely available and is used successfully in conjunction with a reusable ureteroscope. A hybrid strategy in flexible ureteroscopy represents using both instruments, considering the high risk for breakage cases and the economic impact this strategy would imply. It has been shown that large stones located in the lower calyx, a steep infundibulopelvic angle, or abnormal anatomy of the renourinary system such as horseshoe kidney or ectopic pelvic kidney are an unofficial recommendation for using suFURS, protecting the expensive reFURS from breakages. Furthermore, data suggest that the more flexible ureteroscopies are performed, the better the economic outcomes are provided when using exclusively reFURS. Consequently, adopting a hybrid strategy in managing renal stones through flexible ureteroscopy also has a favorable impact on the economic management of such patients.
